# Neuron-specific antioxidant OXR1 extends survival of a mouse model of amyotrophic lateral sclerosis

**DOI:** 10.1093/brain/awv039

**Published:** 2015-03-09

**Authors:** Kevin X. Liu, Benjamin Edwards, Sheena Lee, Mattéa J. Finelli, Ben Davies, Kay E. Davies, Peter L. Oliver

**Affiliations:** 1 Medical Research Council Functional Genomics Unit, Department of Physiology, Anatomy and Genetics, University of Oxford, Parks Road, Oxford OX1 3QX, UK; 2 Wellcome Trust Centre for Human Genetics, Roosevelt Drive, Oxford OX3 7BN, UK

**Keywords:** neurodegeneration, inflammation, oxidative stress, ALS, motor neuron disease

## Abstract

Amyotrophic lateral sclerosis is a devastating neurodegenerative disorder characterized by the progressive loss of spinal motor neurons. While the aetiological mechanisms underlying the disease remain poorly understood, oxidative stress is a central component of amyotrophic lateral sclerosis and contributes to motor neuron injury. Recently, oxidation resistance 1 (OXR1) has emerged as a critical regulator of neuronal survival in response to oxidative stress, and is upregulated in the spinal cord of patients with amyotrophic lateral sclerosis. Here, we tested the hypothesis that OXR1 is a key neuroprotective factor during amyotrophic lateral sclerosis pathogenesis by crossing a new transgenic mouse line that overexpresses OXR1 in neurons with the SOD1^G93A^ mouse model of amyotrophic lateral sclerosis. Interestingly, we report that overexpression of OXR1 significantly extends survival, improves motor deficits, and delays pathology in the spinal cord and in muscles of SOD1^G93A^ mice. Furthermore, we find that overexpression of OXR1 in neurons significantly delays non-cell-autonomous neuroinflammatory response, classic complement system activation, and STAT3 activation through transcriptomic analysis of spinal cords of SOD1^G93A^ mice. Taken together, these data identify OXR1 as the first neuron-specific antioxidant modulator of pathogenesis and disease progression in SOD1-mediated amyotrophic lateral sclerosis, and suggest that OXR1 may serve as a novel target for future therapeutic strategies.

## Abstract (precis)

Oxidative stress is a key factor contributing to motor neuron injury in amyotrophic lateral sclerosis (ALS). Liu *et al.* show that overexpression of *oxidation resistance 1* (*Oxr1*) in neurons reduces pathology and extends lifespan in an ALS mouse model. Manipulation of OXR1 levels may have therapeutic benefit in neurodegenerative disease.

## Graphical abstract

**Figure:**
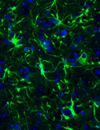
Oxidative stress is a key factor contributing to motor neuron injury in amyotrophic lateral sclerosis (ALS). Liu *et al.* show that overexpression of *oxidation resistance 1* (*Oxr1*) in neurons reduces pathology and extends lifespan in an ALS mouse model. Manipulation of OXR1 levels may have therapeutic benefit in neurodegenerative disease.

## Introduction

The neurodegenerative disorder amyotrophic lateral sclerosis (ALS) is characterized by the progressive loss of upper motor neurons in the cortex, and lower motor neurons in the brainstem and the spinal cord, resulting in severe muscle wasting and death due to respiratory failure (Hardiman *et al.*, 2011). While most ALS cases are sporadic, ∼10% of cases are familial with mutations in Cu/Zn super oxide dismutase (SOD1) accounting for 20% of familial ALS cases (Ferraiuolo *et al.*, 2011). Many studies have identified oxidative stress as a feature of ALS pathogenesis; for example, increased production of reactive oxygen species (ROS) as well as oxidative damage to proteins and lipids have been described in patients (Barber and Shaw, 2010; D'Amico *et al.*, 2013). Although the pathways that instigate oxidative stress in ALS have not been elucidated, it has been postulated that the close relationship of SOD1 to mitochondrial function influences the generation of reactive oxygen species (Valentine *et al.*, 2005; Vehvilainen *et al.*, 2014). Indeed, patients with familial SOD1 ALS show pathological features indicative of oxidative stress, such as increased 3-nitrotyrosine levels, a marker for oxidative damage in motor neurons (Beal *et al.*, 1997). Transgenic mice overexpressing human SOD1^G93A^ recapitulate many clinical features of ALS patients, including defects in motor control, muscle denervation, and progressive degeneration of motor neurons (Gurney *et al.*, 1994). These SOD1^G93A^ mice and other ALS mouse models have been particularly valuable for uncovering presymptomatic and early symptomatic pathogenic pathways, including excitotoxicity, protein aggregation, and neuroinflammation (Ferraiuolo *et al.*, 2011; Swarup and Julien, 2011). Furthermore, SOD1 mouse mutants have provided additional evidence that oxidative stress and mitochondrial dysfunction are detrimental to motor neuron survival *in vivo* (Valentine *et al.*, 2005; Barber and Shaw, 2010; Ferraiuolo *et al.*, 2011; Swarup and Julien, 2011; Pollari *et al.*, 2014). However, despite these new insights, the mechanisms by which ALS manifests in patients remain unclear (Ferraiuolo *et al.*, 2011; Swarup and Julien, 2011).

Initially identified in a screen for genes that protect against oxidative stress-induced damage, oxidation resistance 1 (*Oxr1*) is a highly conserved gene expressed throughout the CNS, and confers neuronal sensitivity to oxidative stress-mediated apoptosis (Volkert *et al.*, 2000; Elliott and Volkert, 2004; Oliver *et al.*, 2011). Specifically, loss of *Oxr1* in mice leads to severe cerebellar ataxia, oxidative damage and cell death in cerebellar granule neurons, and death before postnatal Day 26 (P26) (Oliver *et al.*, 2011). Although OXR1 does not possess antioxidant scavenging properties, overexpression of OXR1 renders neurons less susceptible to exogenous peroxide-induced apoptosis *in vitro*, suggesting that OXR1 regulates downstream pathways to protect neurons from oxidative stress damage (Oliver *et al.*, 2011). Interestingly, intermediate isoforms of OXR1 are upregulated in the spinal cord of ALS patients, and the full-length isoform of OXR1 is upregulated in the presymptomatic low-copy SOD1^G93A^ ALS mice. These findings provide the first line of evidence that overexpression of OXR1 may not only act as an early marker of oxidative stress, but also have neuroprotective properties during neurodegenerative disease progression (Oliver *et al.*, 2011).

Here, we demonstrate that overexpression of OXR1 in neurons extends survival of SOD1^G93A^ ALS mice and delays spinal cord and muscle pathogenesis. Moreover, OXR1 overexpression delays early SOD1^G93A^ ALS-induced alterations to the transcriptome, including the activation of neuroinflammatory pathways in the spinal cord. Taken together, we reveal therapeutic potential of OXR1 in modulating ALS.

## Materials and methods

### Animals

All experiments were conducted in adherence to the guidelines set forth by the UK Home Office regulations, and with the approval of the University of Oxford Ethical Review Panel. Tg(*Prnp-Oxr1*) mice, overexpressing an HA-tagged *Oxr1* transgene driven by the mouse *Prnp* promoter, were generated as previously described (Oliver *et al.*, 2011) and maintained on a C57BL/6 background. Transgenic SOD1^G93A^ mice were originally derived from the B6SJL-TgN(SOD1-G93A)1Gur (Jackson Laboratory) strain and maintained on a C57BL/6 background. All mice studied were the progeny of a SOD1^G93A^ male and a Tg(*Prnp-Oxr1*) female. Mice from several litters were required to generate each experimental cohort; for quantitative transcriptional, pathological and immunohistochemical analyses, all cohorts were sex-matched and a maximum of one animal per genotype was used from a single litter. The *bella* mutant mouse, a progeny of an *N*-ethyl-*N*-nitrosourea-injected BALB/c male and a C3H/HeH female, has been described previously (Oliver *et al.*, 2011). The day of vaginal plug and the day of birth were designated as E0.5 and P0, respectively.

### Disease course analysis and behaviour tests

Disease onset was retrospectively defined as the age when mice reached maximum body weight as previously described (Weydt *et al.*, 2003; Boillee *et al.*, 2006; Ludolph *et al.*, 2007). Disease end-stage was defined by the age when mice suffered from functional paralysis of both hindlimbs; this phenotype has recently been established experimentally as an earlier and more humane but predictable and reproducible endpoint for transgenic mouse models of ALS, limiting the duration of disease exposure (Solomon *et al.*, 2011). Disease progression was retrospectively defined as the number of days between disease onset and disease end-stage for each mouse.

To test motor function, mice were placed on a grooved plastic beam of a Rotarod device (Ugo Basile), which revolves at a default 5 rpm, facing in an orientation opposite to the rotation. The time latency to fall from the rod or complete two rotations on the rod without an attempt to run was recorded; a single trial was carried out per day over 3 days in total at each experimental time point and the recorded values were averaged. To test for muscle strength, mice were held by their tails, and gently placed on a grip strength device (Chatillon), such that their forepaws gripped a metal mesh bar attached to the apparatus. The mice were then gently pulled across the metal mesh bar, and maximum force (*g*) exerted before the mice released their grip was recorded. Three trials were recorded and the average force was calculated. All behavioural tests were conducted with the experimenter blind to genotypes.

### Immunohistochemistry and histology

Mice were transcardially perfused with 0.9% saline containing 10 U/ml heparin, followed by 4% paraformaldehyde (PFA). Brains and spinal cords were dissected and post-fixed overnight in 4% PFA at 4°C. Tissue was cryoprotected with 30% sucrose, mounted in O.C.T. (VWR) medium, and sectioned at 15 μm on a cryostat (Leica). For muscle fibre type staining, tissue samples were freshly dissected and frozen in O.C.T. on isopentane in dry ice. Frozen transverse sections were cut at 10 μm on a cryostat (Leica). Sections were blocked in 5% bovine serum albumin (BSA) (Sigma)/PBS/0.3% Triton^TM^ X100 at room temperature for 1 h. Incubation with primary antibodies was conducted overnight (16 h) at 4°C or for 1 h at room temperature. For GFAP immunohistochemistry, antibody incubation was carried out for 26 h at 4°C. After washing with phosphate-buffered saline (PBS), sections were incubated with appropriate Alexa Fluor® secondary antibodies diluted 1:500 (Invitrogen) for 1 h at room temperature, washed in PBS and mounted in Histomount (National Diagnostics) or Vetashield with DAPI (Vector Laboratories).

The following primary antibodies and dilutions were used: rat anti-CD68 (1:400, Millipore Bioscience Research Reagents); rabbit anti-GFAP (1:500, ab7260, Abcam); rabbit anti-HA (1:100, Sigma); rabbit anti-HO-1 (1:300, Abcam); mouse anti-MYHC1 (1:200, B8-F8, German Collection of Microorganisms and Cell Cultures); mouse anti-MYHC2A (1:200, SC-71, German Collection of Microorganisms and Cell Cultures); mouse IgM anti-MYHC2B (1:100, BF-F3, German Collection of Microorganisms and Cell Cultures); and mouse anti-NeuN (1:500, Millipore).

For neuromuscular junction staining, fresh dissected muscles were teased apart, and placed in 10% foetal calf serum in PBS/0.2 Triton^TM^ X-100 with an appropriate dilution of Alexa Fluor® 488 conjugated α-bungarotoxin (Invitrogen) overnight at 4°C. The teased muscle samples were subsequently washed in PBS three times, before being further teased apart, placed on a slide, and mounted using Histomount.

For motor neuron counts, all large ventral horn motor neurons on eight matched Nissl-stained 15-μm sections of lumbar spinal cord segments (L1-L6) were counted and averaged per section. For quantitative immunohistochemistry, six matched 15-μm sections of lumbar spinal cord segments (L1-L6) per animal were stained and 0.2 mm^2^ regions of each anterior horn were acquired using an AxioCam HR digital camera (Zeiss) and quantified for total signal intensity using ImageJ software. For each antibody, all slides were processed in parallel. All quantification was conducted with the experimenter blind to the genotypes.

### Muscle histopathology

For haematoxylin and eosin stain, fresh muscle sections were placed in haematoxylin for an appropriate time, no longer than 15 min, and briefly washed in distilled water. Sections were subsequently bled in 70% ethanol/0.1% hydrochloric acid for 10 s, and washed in distilled water. The sections were then placed in eosin for 2 min, washed in distilled water, dehydrated and then mounted using Histomount. For muscle atrophy measurements, images were acquired using an AxioCam HR digital camera (Zeiss) and the area of muscle in atrophy and total muscle area were obtained using AxioVision software (Zeiss).

For succinic dehydrogenase staining, fresh muscle sections were incubated in 0.05 M phosphate buffer/0.05 M sodium succinate/0.05% nitro blue tetrazolium chloride for 30 min at 37°C, and subsequently rinsed in PBS. Sections were then fixed in 4% PFA for 5 min and rinsed in 15% ethanol before being mounted using Histomount. All quantification was conducted with the experimenter blind to the genotypes.

### Immunoprecipitation and immunoblotting

Tissue extracts were prepared using appropriate amounts of immunoprecipitation buffer (50 mM Tris-Cl, pH. 7.5, 150 mM NaCl, 1% CHAPS) for immunoprecipitation, or RIPA buffer (50 mM Tris-Cl, pH 8.0, 150 mM NaCl, 0.1% SDS, 1% sodium deoxycholate, 1% NP-40) for immunoblotting. Protein concentrations were quantified using BCA assays (Pierce Thermo Scientific) for equal loading. For immunoprecipitation, tissue extracts were incubated with Sepharose G-beads (Sigma) for 1 h at 4°C, and after spinning down, the supernatant was removed and incubated with an appropriate amount of antibody for 2 h at 4°C. Appropriate amounts of Sepharose G-beads were added to the tissue extract and antibody concoction for another hour at 4°C. The Sepharose G-beads bound to the antibody were then washed three times with immunoprecipitation buffer. After adding 2× Laemmli sample buffer, protein and immunoprecipitation samples were boiled for 5 min at 100°C, separated by 12% SDS-PAGE, and transferred to PVDF membranes (Amersham). Membranes were subsequently blocked with 5% skimmed milk powder in PBS/2% Tween-20 (PBST) for 1 h at room temperature, before incubation with appropriate primary antibody diluted in milk/PBST for either 1 h at room temperature or overnight at 4°C. After washing with PBST, membranes were incubated with the appropriate HRP-conjugated secondary antibodies (1:10 000, Amersham), and blots were developed using ECL reagents (Amersham). Quantifications were carried out by calculating expression relative to the loading control using ImageJ software. For immunoblotting in Supplementary Fig. 1B and C, blots were processed using ImageQuant Las 4000 (GE Healthcare).

The following primary antibodies and dilutions were used: rabbit anti-HA (1:10 000, Sigma); rabbit anti-HO-1 (1:1000 Enzo Life Sciences); rabbit anti-Oxr1 (1:1000); sheep anti-SOD1 (1:1000, Calbiochem); rabbit anti-STAT3 (1:2000, Cell Signaling); rabbit anti-p-STAT3 (1:1000, Cell Signaling); and mouse anti-β-actin (1:6000, Sigma).

### Microarray analysis

Total RNA was purified from tissue samples from four animals of each genotype using the RNeasy® Mini kit (Qiagen) and contaminating genomic DNA was removed using RNase-free DNase I (Qiagen). The RNA integrity of all samples was assessed on a BioAnalyzer; all samples had a RNA Integrity Number (RIN) ≥8 (Agilent Laboratories). Labelled sense single-stranded DNA for hybridization was generated from 200 ng of RNA with the Affymetrix GeneChip WT PLUS Reagent Kit. The single-stranded DNA was then fragmented and labelled using the Affymetrix WT Terminal Labelling and Controls Kit, and the distribution of fragment lengths was measured on the Agilent BioAnalyzer. The labelled and fragmented single-stranded DNA was hybridized to the Affymetrix GeneChip Mouse Gene 1.0 ST Array, and then washed and stained using the Affymetrix Hybridization, Wash, and Stain Kit, according to the manufacturer’s instructions. Chips were processed on an Affymetrix GeneChip Fluidics Station 450 and Scanner 3000.

Microarray data were Robust Multi-array Average (RMA) normalized using GeneSpring GX12.6 (Agilent). Differentially expressed genes were identified using a false discovery rate of ≤0.05 with a Benjamini and Hochberg multiple testing correction (limma). The genes that were designated as ‘rescued’ fulfilled the following criteria: (i) genes that had a >1.3-fold change and *P* < 0.05 between SOD1^G93A^ samples and both wild-type and Tg(*Prnp-Oxr1*) samples; and (ii) genes that had a <1.2-fold change or *P* > 0.05 between SOD1^G93A^/Tg(*Prnp-Oxr1*) samples and both wild-type and Tg(*Prnp-Oxr1*) samples. The Ingenuity Pathways Analysis tool (Ingenuity Systems, www.ingenuity.com) was used to gain additional *in silico* functional information. The Functions tool in Ingenuity relates genes of interest to known biological functions and disease states and was used to detect the most significant functional pathways in the ‘rescued’ genes. Microarray data are available on request.

### Quantitative real time PCR

Complementary DNA (cDNA) was synthesized with the RevertAid^TM^ First Strand cDNA Synthesis Kit (Fermentas), using 1 μg of total RNA for each reaction. Quantitative RT-PCR reactions were subsequently carried out using SYBR Green PCR master mix (Applied Biosystems) with primers and cDNA added in optimized concentrations, using a StepOne real-time PCR machine (Applied Biosystems) with cycling conditions at 95°C for 10 s, followed by 40 cycles of 55°C for 15 s, 60°C for 10 s. Gene amplification specificity was verified by melting curve analyses. The primer sequences used: *C1qa* (5'-CAACGTGGTTATCTTTGACAAGGT-3' and 5'-GAAGTTGAAGTAATAGAAGCCGGG-3'); *C1qb* (5'-CACCAACGCGAACGAGAACT-3' and 5'-GGCCAGGCACCTTGCA-3'); *C1qc* (5'-CTACTTCGTCTACTACACATCGCA-3' and 5'-CACCATGCCATTGTAGTCATTGAC-3'); *Cd52* (5'-TCCTCCTCTTCCTCACTATCATTCT-3' and 5'-GGCACATTAAGGTATTGGCAAAGA-3'); *Cd68* (5'-CTACATCAGAGCCCGAGTAC -3' and 5'-CTGGTAGGTTGATTGTCGTCTG-3'); *Ctss* (5'-TACCAGGGTTCTTGTGGTGC-3' and 5'-AGGGATATCAGCTTCCCCGT-3'); *Gapdh* (5'-GCTACACTGAGGACCAGGTTGTC-3' and 5'-AGCCCCGGCATCGAA-3'); *hSOD1* (5'-GGCCAAAGGATHAAGAGAGGC-3' and 5'-TGTGCGGCCAATGATGCAAT-3'); *Lyz2* (5'-TCCTGACTCTGGGACTCCTC-3' and 5'-AGCCAGCCATTCCATTCCTT-3'); *Mpeg1* (5'-AGAAACCGGATCTACACAGTGAAA-3' and 5'-GATTACGTGTGTGCCATAGTTGAG-3'); *Oxr1* (5'-CAGTCGTGACTGGACAGGTTT-3' and 5'-ATGGGCTACATCTGGAGTCG-3'); *Serpina3n* (5'-CGAAACTGTACCCTCTGACTGTAT-3' and 5'-TTGGCTATCTTGGCTATAAAGGGG-3'); and *Vim* (5'-CGGAAAGTGGAATCCTTGCAGG-3' and 5'-AGCAGTGAGGTCAGGCTTGGAA-3'). All values obtained were normalized with respect to mRNA levels of *Gapdh*.

### Statistical analysis

Results were analysed using Prism (GraphPad Software, Inc.) with the statistical tests described in the text. Data are presented as mean ± SEM (standard error of mean), with *n* indicating the number of independent biological replicates used in each group for comparison.

## Results

### Tg(*Prnp-Oxr1*) transgenic mice overexpress OXR1 specifically in neurons

**Figure 1:**
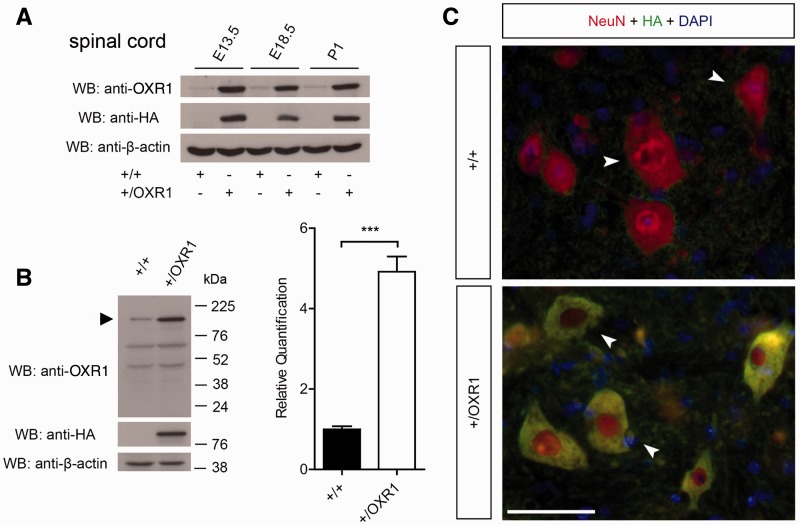
**Tg(*Prnp-Oxr1*) mice overexpress OXR1 specifically in neurons.** (**A**) *Prnp*-promoter drives overexpression of HA-tagged full-length OXR1 as early as embryonic Day 13.5 (E13.5) in the spinal cord as shown by western blot in Tg(*Prnp-Oxr1*) mice (+/OXR1) when compared with wild-type (+/+). (**B**) Representative western blot showing a 5-fold increase in spinal cord expression of OXR1 (*arrow*) in +/OXR1 mice when compared with wild-type. The same membrane was reprobed with anti-HA to show HA-tagged full-length OXR1 and anti-β-actin as the loading control for quantification; values are mean ± SEM (*n* = 3 per genotype; ***P < 0.001, two-tailed Student’s *t*-test). Sizes of the bands correspond to those in (**A**). (**C**) Immunohistochemical staining for NeuN, a marker for neurons (*arrows*), and HA-tagged OXR1, in the spinal cord, demonstrating that *Prnp*-promoter driven OXR1 overexpression in +/OXR1 mice is specific to neurons. Scale bar = 50 µm. P1 = postnatal Day 1; WB = western blot.

OXR1 is highly expressed in the CNS, and specifically in neurons (Supplementary Fig. 1A) (Oliver *et al.*, 2011). The detection of *OXR1* transcripts has also been reported outside of the CNS (Yang *et al.*, 2014); however, by western blot we show here that the brain and spinal cord express the majority of the full-length OXR1 protein, with multiple smaller isoforms present in the CNS and other organs (Supplementary Fig. 1B). Thus, to study whether overexpression of OXR1 is neuroprotective *in vivo*, we generated a transgenic mouse [Tg(*Prnp-Oxr1*)], in which the mouse prion protein *Prnp* promoter drives overexpression of a full-length mouse *Oxr1* cDNA with a C-terminal HA-tag (Borchelt *et al.*, 1996). First we confirmed that expression of HA-tagged OXR1 protein occurs in the spinal cord and brain (Supplementary Fig. 1C); in addition, the *Oxr1* transgene is overexpressed as early as embryonic Day 13.5 (Fig. 1A and Supplementary Fig. 1D) and adult transgenic Tg(*Prnp-Oxr1*) mice show a consistent 5-fold increase in OXR1 protein expression compared to wild-type controls in the spinal cord (Fig. 1B) and brain (Supplementary Fig. 1E). Immunohistochemistry confirmed the neuron-specific overexpression of OXR1; all Tg(*Prnp-Oxr1*) neurons express cytoplasmic HA-tagged OXR1, and no HA-stained cells are found in any NeuN-negative cell populations in the brain or spinal cord (Fig. 1C and Supplementary Fig. 1F).

### OXR1 overexpression in neurons extends survival in SOD1^G93A^ mice

As we showed previously that OXR1 is upregulated in the spinal cord of ALS patients (Oliver *et al.*, 2011), we investigated whether overexpression of OXR1 from early development can delay or prevent motor neuron degeneration by crossing Tg(*Prnp-Oxr1*) mice with SOD1^G93A^ mice (Gurney *et al.*, 1994). We analysed offspring from this cross of all four genotypic combinations [wild-type Tg(*Prnp-Oxr1*) (+/OXR1), SOD1^G93A^ (SOD/+), and SOD/OXR1] for longitudinal behavioural and pathological parameters. First, we confirmed that expression of the mutant SOD1^G93A^ and *Oxr1* transgenes were stable, irrespective of genotype, by immunoblotting and quantitative RT-PCR (Supplementary Fig. 1G–J). Importantly, when we compared wild-type mice with +/OXR1 progeny from the same cross, we observed no overt muscle or neuromuscular junction pathology (Supplementary Fig. 2A and B). Consistent with these data, +/OXR1 mice do not perform differently from wild-type controls on Rotarod or grip strength tests (Supplementary Fig. 2C–F), and there is no difference in survival rates (data not shown). Together, these data confirm that no detrimental pathology occurs under neuronal overexpression of OXR1 *in vivo*.

**Figure 2:**
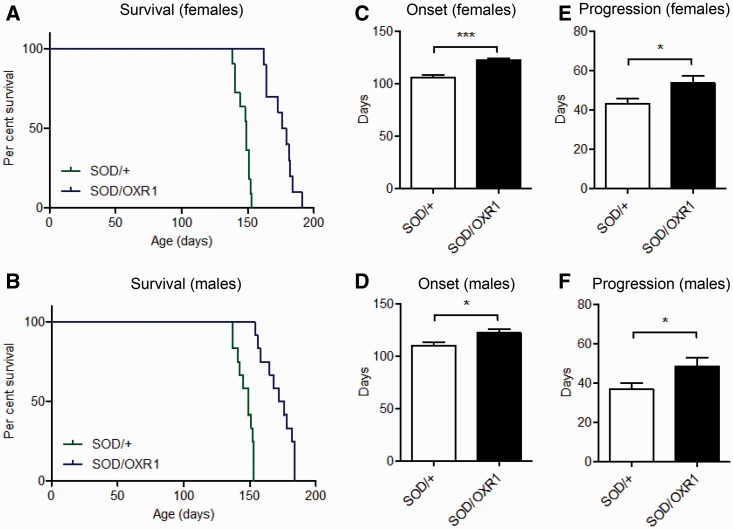
**Neuronal overexpression of OXR1 extends lifespan of SOD1^G93A^ mice.** Kaplan-Meier log rank test for survival, showing OXR1 overexpression increases median survival (**A**) from 149 days for SOD/+ females to 178 days for SOD/OXR1 females; and (**B**) from 149 days for SOD/+ males to 174 days for SOD/OXR1 males (*n* = 10–12 per sex per genotype). (**A** and **B**) Survival curves are significantly different by Mantel-Cox test, *P* < 0.0001 (χ^2^ = 24.99) for males, and *P* < 0.0001 (χ^2^ = 22.05) for females. OXR1 overexpression delays disease onset, defined by the age of maximum body weight, (**C**) from 106 days for SOD/+ females to 122 days for SOD/OXR1 females; and (**D**) from 110 days for SOD/+ males to 122 days for SOD/OXR1 males. OXR1 overexpression slows disease progression, defined by time of disease onset to end-stage, (**E**) from 43 days for SOD/+ females to 53 days for SOD/OXR1 females; and (**F**) from 37 days for SOD/+ males to 48 days for SOD/OXR1 males. (**C**–**F**) Values are mean ± SEM (*n* = 9–16 per sex per genotype; *P < 0.05, **P < 0.01, ***P < 0.001; two-tailed Student’s test).

We also obtained survival rates in parallel for SOD/+ and SOD/OXR1 mice using functional paralysis of both hindlimbs to determine disease end-stage (Solomon *et al.*, 2011). Strikingly, in females, median survival is significantly increased by 19% (*P* < 0.0001), from 149 days for SOD/+ mice to 178 days for SOD/OXR1 animals (Fig. 2A). Similarly, in males, median survival is significantly increased by 16.8% (*P* < 0.0001), from 149 days for SOD/+ mice to 174 days for SOD/OXR1 mice (Fig. 2B). We also objectively determined disease onset by assessing peak weight (Ludolph *et al.*, 2007); interestingly, disease onset is significantly delayed by 15.6% (*P* < 0.0001) in females, from 106 days for SOD/+ mice to 122 days for SOD/OXR1 animals, and by 11.0% (*P* < 0.05) in males, from 110 days for SOD/+ mice to 122 days for SOD/OXR1 animals (Fig. 2C and D). Furthermore, disease progression is significantly extended by 24.2% (*P* < 0.05) in females, from 43 days for SOD/+ mice to 53 days for SOD/OXR1 animals, and by 32.2% (*P* < 0.05) in males, from 37 days for SOD/+ mice to 48 days for SOD/OXR1 animals (Fig. 2E and F). These data demonstrate that neuron-specific OXR1 overexpression improves survival in SOD^G93A^ mice.

### Neuronal OXR1 overexpression improves motor function and spinal cord pathology in SOD1^G93A^ mice

**Figure 3:**
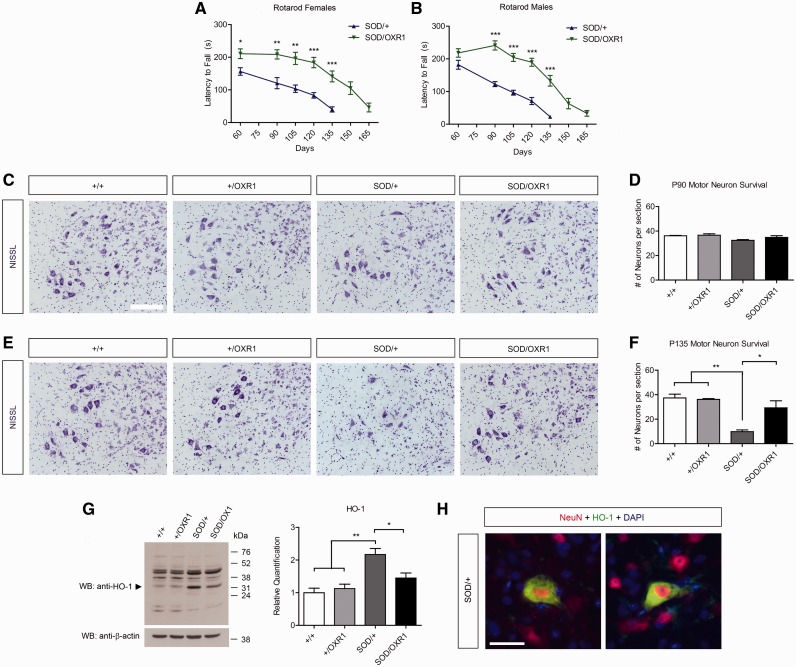
**Neuronal overexpression of OXR1 improves motor function and motor neuron survival in SOD1^G93A^ mice.** (**A**) SOD/OXR1 females and (**B**) males have improved motor performance on the accelerating Rotarod when compared with SOD/+ females and males, respectively. (**A** and **B**) Values are mean ± SEM of motor performance (seconds, s) for mice still alive at each respective time point (*n* = 9–11 per sex per genotype at Days 60–120 and *n* = 6–11 per sex per genotype at Days 135–165 due animals reaching end-stage; *P < 0.05, **P < 0.01, ***P < 0.001, Mann-Whitney U-tests). Representative images of Nissl-stained motor neurons in matching lumbar spinal cord cross-sections at Day 90 (**C**) and Day 135 (**E**). Motor neuron survival counts in lumbar spinal cord cross-sections at Day 90 (**D**) and Day 135 (**F**); values are mean ± SEM (*n* = 3–4 per genotype; *P < 0.05, **P < 0.01; 1-way ANOVA, with Tukey’s *post hoc* tests). Scale bar = 100 µm. (**G**) Western blot showing reduced induction of HMOX1 (HO-1, *arrow*) in SOD/OXR1 mice. The same membrane was re-probed with anti-β-actin as the loading control for quantification; values are mean ± SEM (*n* = 3 per genotype; *P < 0.05, **P < 0.01; one-way ANOVA, with Tukey’s *post hoc* tests). (**H**) Immunohistochemical staining for HMOX1 and NeuN in the spinal cord demonstrating HMOX1 expression in neurons. Scale bar = 30 µm. P90 = postnatal Day 90; P135 = postnatal Day 135; WB = western blot.

Given the significant extension of lifespan in SOD/OXR1 mice, we next determined neuroprotective effects of OXR1 overexpression by comparing behaviour and pathology of SOD/+ mice to that of SOD/OXR1 mice at the same time-points during ALS disease pathogenesis, a method commonly used in studies examining genetic modifiers of SOD1-mediated ALS (Boillee *et al.*, 2006; Yamanaka *et al.*, 2008; Hetz *et al.*, 2009; Van Hoecke *et al.*, 2012; Frakes *et al.*, 2014; Seijffers *et al.*, 2014). First, to examine whether neuronal overexpression of OXR1 improves motor dysfunction in SOD1^G93A^ mice, we measured Rotarod performance at multiple time points. Interestingly, average latency to fall for SOD/OXR1 mice is significantly longer than that of SOD/+ mice at all time points, beginning as early as postnatal Day 60 for females (*P* < 0.05) and Day 90 for males (*P* < 0.001) (Fig. 3A and B). We then quantified motor neuron survival in the lumbar spinal cord at Days 90 and 135 to determine whether the improved motor function in SOD/OXR1 mice is a result of delayed neuropathology. Importantly, while no significant differences in motor neuron survival were observed between the four genotypes at Day 90, motor neuron viability is significantly improved in SOD/OXR1 mice when compared to that of SOD/+ mice at Day 135 (*P* < 0.05), with motor neuron numbers in SOD/OXR1 mice almost equivalent to wild-type or +/OXR1 animals (Fig. 3C–F). We previously showed that OXR1 protects against oxidative stress-induced apoptosis (Oliver *et al.*, 2011), therefore we determined whether neuronal overexpression of OXR1 reduces oxidative stress in spinal cord of SOD/+ mice by immunoblotting for heme-oxygenase 1 (HMOX1, previously known as HO-1), a marker of oxidative stress in SOD1^G93A^ mice (Ferrante *et al.*, 1997). HMOX1 expression is significantly induced in SOD/+ mice at Day 135 when compared to wild-type and +/OXR1 controls (*P* < 0.01; Fig. 3G), with this induction detected in neuronal cells of the spinal cord (Fig. 3H). Importantly, HMOX1 induction is significantly reduced in SOD/OXR1 animals when compared to SOD/+ mice (*P* < 0.05; Fig. 3G). Together, these results establish that OXR1 ameliorates motor function and delays pathogenesis in the spinal cord of SOD1^G93A^ mice.

### Neuronal OXR1 overexpression improves muscle function and delays muscle pathology in SOD1^G93A^ mice

**Figure 4:**
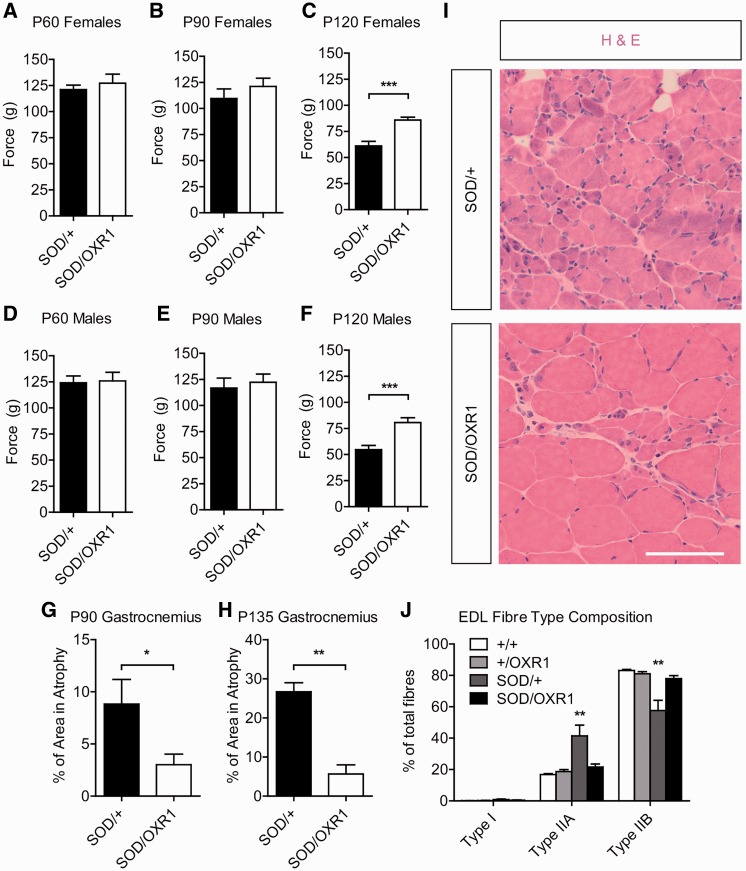
**Neuronal overexpression of OXR1 delays muscle pathology in SOD1^G93A^ ALS mice.** (**A–C**) SOD/OXR1 females show significantly improved muscle strength (*g*) on a grip strength test when compared with SOD/+ females at (**C**) Day 120 (P120), but not at (**A**) Day 60 (P60) or (**B**) Day 90 (P90). (**D**–**F**) SOD/OXR1 males show significantly improved muscle strength (g) on grip strength test when compared with SOD/+ males at (**F**) Day 120, but not at (**D**) Day 60 or (**E**) Day 90. (**A**–**F**) Values are mean ± SEM (*n* = 12–24 per genotype; ***P < 0.001, Mann-Whitney U tests). (**G** and **H**) Gastrocnemius muscle atrophy is decreased in SOD/OXR1 mice compared with SOD/+ mice at (**G**) Day 90 and (**H**) Day 135. (**G** and **H**) Values are mean ± SEM (*n* = 3–6 per genotype; *P < 0.05, **P < 0.01, two-tailed Student’s test). (**I**) Representative images of haemotoxylin and eosin (H&E) stained gastrocnemius muscle fibres at Day 135. (**J**) Extensor digitorum longus (EDL) muscles of SOD/+ mice have increased type IIA and decreased type IIB fibres, when compared to wild-type (+/+), +/OXR1, and SOD/OXR1 mice at Day 90. Values are mean ± SEM (*n* = 4–6 per genotype; **P < 0.01, two-way ANOVA, followed by Bonferroni *post hoc* tests).

As muscle dysfunction and atrophy are downstream consequences of motor neuron loss in ALS patients and SOD1^G93A^ mice (Gurney *et al.*, 1994; Abe *et al.*, 1996), we investigated whether overexpression of OXR1 in neurons improves muscle pathology in SOD1^G93A^ mice. To measure muscle function, we analysed performance of mice on a grip strength monitor, and found that SOD/OXR1 mice have significantly improved muscle strength when compared with SOD/+ animals (*P* < 0.05) from Day 120 in both males and females (Fig. 4A–F). Given gastrocnemius muscle atrophy has been noted in SOD1^G93A^ mice (Gurney *et al.*, 1994), we first conducted histological analysis of this muscle group. Gastrocnemius muscle atrophy of SOD/OXR1 mice is significantly reduced by ∼2-fold at Day 90 (*P* < 0.05) and ∼3-fold at Day 135 (*P* < 0.01), when compared to that of SOD/+ mice (Fig. 4G–I). Interestingly, we also found that abnormal gastrocnemius neuromuscular junction morphology in SOD/+ mice is significantly reduced in SOD/OXR1 animals at Day 90 (*P* < 0.05) (Supplementary Fig. 2G–I). Finally, previous studies reported that extensor digitorum longus muscles of SOD1^G93A^ mice become more fatigue resistant (Kieran *et al.*, 2005), thus we examined muscle fibre-type by immunohistochemical staining for type I, IIA and IIB fibres. We found that EDL muscles of SOD/+ mice have a significant decrease in type IIB fibres, and an increase in type IIA fibres when compared to that of wild-type, +/OXR1 or SOD/OXR1 animals (*P* < 0.01) (Fig. 4J and Supplementary Fig. 2J). Corroborating these findings, staining for oxidative enzyme succinate dehydrogenase showed a marked increase in dark-stained muscle fibres only in EDL muscles of SOD/+ mice (Supplementary Fig. 2K). These data demonstrate that overexpression of OXR1 in neurons delays atrophy in gastrocnemius muscles, and abnormal changes in oxidative capacity in extensor digitorum longus muscles of SOD1^G93A^ mice.

### Transcriptomics reveals that OXR1 overexpression in neurons delays early gene expression changes in the spinal cord of SOD1^G93A^ mice

We then chose to investigate how OXR1 modulates pathogenesis in SOD1^G93A^ mice by performing microarray transcriptome analysis on spinal cord tissue at Day 90 prior to significant motor neuron loss. We purified RNA from the lumbar region of littermate mice of all four genotypic combinations to correspond with the same region used for quantifying motor neuron survival and to ensure that potentially important non-cell autonomous expression changes could be identified (Fig. 3C and E). Comparing the SOD/+ expression profile to that of wild-type and +/OXR1 mice identified 65 differentially expressed genes (Supplementary Table 1), including dysregulation of *Atf3*, *C1qa*, *Ctss*, *HexB*, and *Sprr1a*, confirming results from previous studies of the SOD^G93A^ spinal cord (Olsen *et al.*, 2001; Yoshihara *et al.*, 2002; Perrin *et al.*, 2005; Ferraiuolo *et al.*, 2007; Lobsiger *et al.*, 2007; Chen *et al.*, 2010; Lincecum *et al.*, 2010). Importantly, we also observed no significant gene expression changes between wild-type and +/OXR1 mice apart from the expected *Oxr1* overexpression from the *Oxr1* transgene itself (Supplementary Table 2); this further confirms that presence of the *Oxr1* transgene does not have a detrimental effect on spinal cord function or transcriptional regulation, in-line with behavioural and pathological data described above (Supplementary Figs 1 and 2).

**Figure 5:**
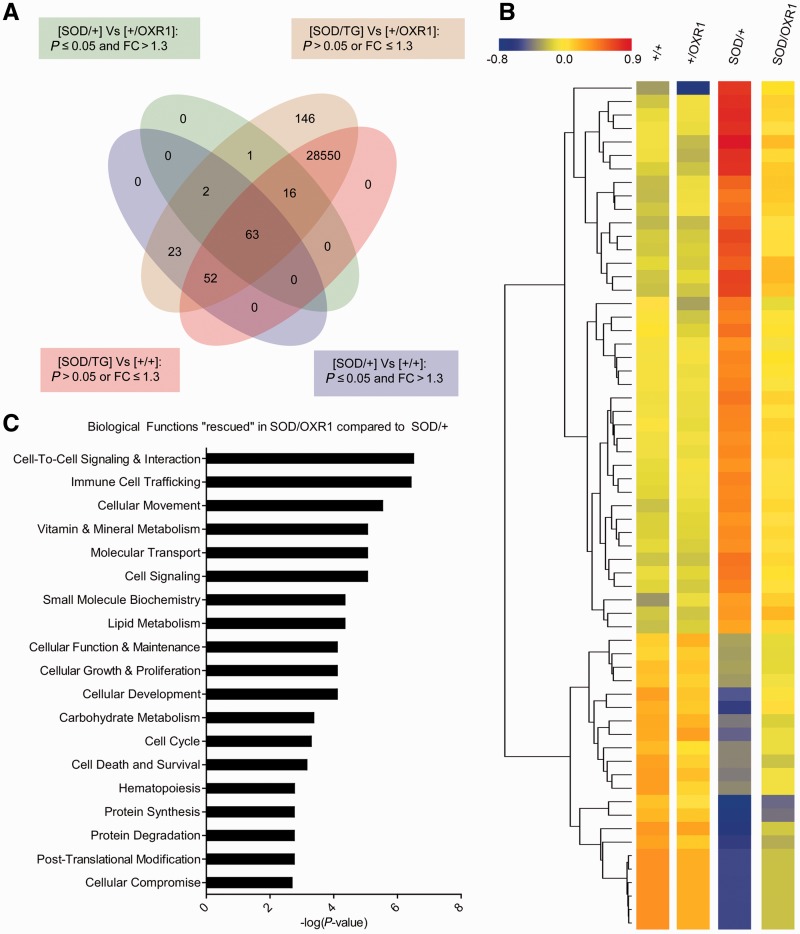
**Neuronal OXR1 overexpression delays early transcriptome changes in SOD1^G93A^ spinal cord.** (**A**) Venn diagram illustrates the common expression overlap from microarray analysis to identify 63 ‘rescued’ genes; those that are significantly changed (*P* ≤ 0.05) by >1.3-fold in SOD/+ spinal cord, but not significantly changed (*P* > 0.05) or by <1.2-fold in the SOD/OXR1 spinal cord at Day 90 after correcting for multiple comparisons. (**B**) Heat map of normalized signal intensity values for all genes identified as ‘rescued’ by neuronal OXR1 overexpression at Day 90. (**C**) Pathway analysis demonstrates that neuronal OXR1 overexpression delays SOD1^G93A^-induced changes in diverse molecular pathways at Day 90. Significance of identified pathways is presented as a –log(*P*-value), where *P* ≤ 0.05 is –log(*P*-value) ≥ 1.3.

More importantly, we identified 63 ‘rescued’ genes in SOD/OXR1 mice; defined as those genes that are significantly altered by >1.3-fold in the SOD/+ spinal cord, but not significantly changed or altered by <1.2-fold in SOD/OXR1 tissue (Fig. 5A and B, and Supplementary Table 3). Using pathway analysis of these genes, we found that overexpression of OXR1 influences a range of important pathogenic mechanisms that have been previously found to be deregulated in ALS (Fig. 5C) (Olsen *et al.*, 2001; Yoshihara *et al.*, 2002; Perrin *et al.*, 2005; Ferraiuolo *et al.*, 2007; Chen *et al.*, 2010; Lincecum *et al.*, 2010). Furthermore, selected genetic modifiers in these particular pathways can extend survival of mutant SOD1 mice (Turner and Talbot, 2008; Riboldi *et al.*, 2011). Together, these data suggest that OXR1 functions upstream of diverse biological functions during ALS pathogenesis and delays early mutant SOD1-mediated alterations to the transcriptome.

### Neuronal OXR1 overexpression modulates neuroinflammatory response in SOD1^G93A^ mice

**Figure 6:**
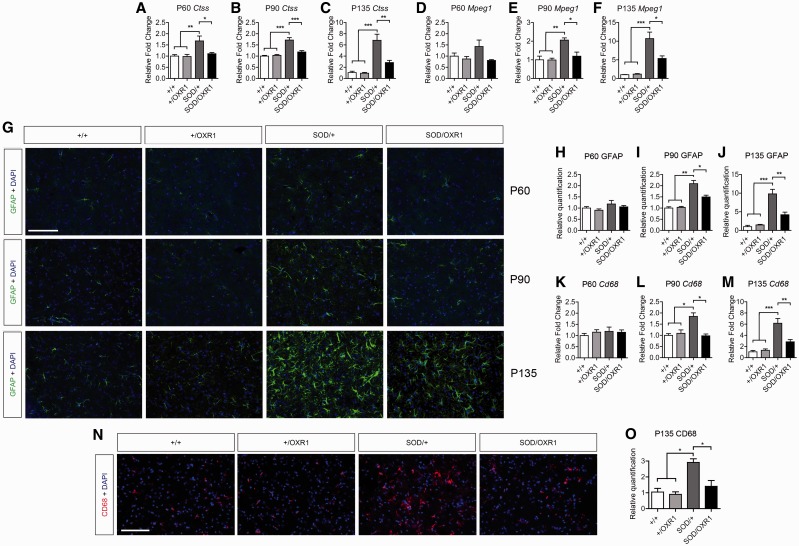
**Neuronal OXR1 overexpression decreases neuroinflammation in SOD1^G93A^ spinal cord.** (**A**–**C**) SOD1^G93A^-induced expression of *Ctss*, a marker of neuroinflammation, in the spinal cord is reduced in SOD/OXR1 mice at Days 60, 90 and 135 as shown by quantitative RT-PCR. (**D**–**F**) Induced expression of *Mpeg1*, a macrophage marker, in the SOD1^G93A^ spinal cord is reduced by overexpression of OXR1 at Day 90 (P90), and Day 135 (P135) as shown by quantitative RT-PCR. (**G**–**J**) Immunohistochemical staining for GFAP on matching sections of lumbar spinal cord, showing significantly decreased astrogliosis in SOD/OXR1 mice when compared to SOD/+ mice at Days 90 and 135. (**K**–**M**) Induced expression of *Cd68,* a marker of microgliosis, in the SOD^G93A^ spinal cord is significantly reduced in SOD/OXR1 mice at Days 90 and 135 as shown by quantitative RT-PCR. (**N**–**O**) Immunohistochemical staining for CD68 on matching sections of lumbar spinal cord shows decreased microgliosis in SOD/OXR1 mice when compared to SOD/+ mice at Day 135. Values are mean ± SEM [*n* = 3–5 per genotype (**A**–**F** and **K**–**M**), *n* = 3 per genotype (**G**–**J** and **N**–**O**); *P < 0.05, **P < 0.01, ***P < 0.001, one-way ANOVA with Tukey’s *post hoc* tests]. Scale bars = 100 µm.

Because immune cell trafficking was identified as a key pathogenic pathway delayed by OXR1 overexpression (Fig. 5C), and the fly orthologue of OXR1 has been implicated in regulating innate immunity (Wang *et al.*, 2012), we chose to further validate four markers of macrophages or immune activation, *Ctss*, *Mpeg1*, *Lyz2*, and *Cd52* (Olsen *et al.*, 2001; Ferraiuolo *et al.*, 2007) (Supplementary Table 3). We examined expression profiles of these genes in independent samples of spinal cord of Days 60, 90 and 135 to assess the extent and timing of candidate gene deregulation both prior to and during disease progression. Quantitative RT-PCR confirmed that *Ctss* and *Lyz2* are significantly upregulated in the spinal cord of SOD/+ mice but not in that of SOD/OXR1 animals at Days 90 and 135 (Fig. 6A–C and Supplementary Fig. 3A–C). Importantly, the same pattern of ‘rescued’ expression for both genes was observed at Day 60, suggesting that OXR1 influences immune pathways prior to disease onset (Fig. 6A and Supplementary Fig. 3A). Induction of *Cd52* and *Mpeg1* is observed in the SOD/+ spinal cord, but not in the SOD/OXR1 spinal cord at Days 90 and 135 (Fig. 6D–F and Supplementary Fig. 3D–F). At Day 135, this effect is more pronounced; for example, relative expression of *Ctss* is increased in the SOD/+ and SOD/OXR1 spinal cord by ∼7-fold and 3-fold, respectively (Fig. 6C).

To further investigate this phenomenon, we conducted quantitative immunohistochemical staining at Days 60, 90 and 135 for GFAP, a marker for astrogliosis (Fig. 6G) (Lincecum *et al.*, 2010; Papadeas *et al.*, 2011). Levels of GFAP-positively stained activated astrocytes are not significantly different at Day 60 across all four genotypes, but by Day 90 a significant 2-fold relative increase in the SOD/+ spinal cord was observed (Fig. 6H and I). At Day 135, while relative GFAP-staining is increased by ∼9-fold in SOD/+ mice compared to wild-type and +/OXR1 animals, expression is significantly reduced in SOD/OXR1 animals close to the levels observed in controls (Fig. 6J). We also investigated the role of OXR1 overexpression on microgliosis in SOD^G93A^ mice, as suggested by the presence of *Cd68* as one of the genes ‘rescued’ in SOD/OXR1 animals (Supplementary Table 3) (Lincecum *et al.*, 2010; Papadeas *et al.*, 2011). First we validated that *Cd68* was significantly upregulated in the spinal cord of SOD/+ mice, but not in SOD/OXR1 animals at Day 90 by quantitative PCR (Fig. 6K and L). Furthermore, at Day 135, *Cd68* was induced by ∼6-fold in SOD/+ mice but was markedly and significantly reduced in SOD/OXR1 animals (Fig. 6M). By quantitative immunohistochemistry, no differences in the levels of CD68-postive activated microglia in the spinal cord were observed between genotypes at Days 60 and 90 (data not shown), but we confirmed that SOD/OXR1 mice have significantly decreased levels of CD68-postive microglia when compared to SOD/+ animals at Day 135 (Fig. 6N and O). Finally, we confirmed that these findings are due to neuron-specific overexpression of OXR1 by demonstrating that overexpression of OXR1 does not co-localize with GFAP-positive activated astrocytes or CD68 activated microglia in SOD/OXR1 mice (Supplementary Fig. 4A and B).

**Figure 7:**
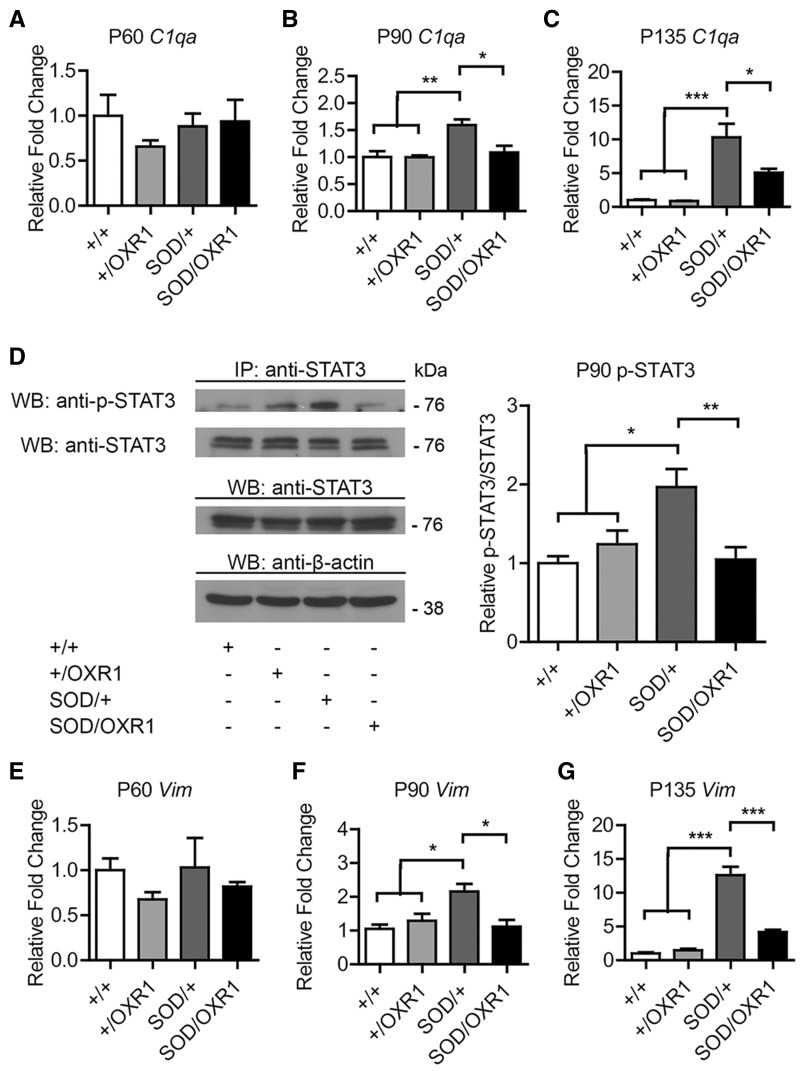
**Neuronal OXR1 overexpression delays activation of neuroinflammatory pathways in the spinal cord of SOD1^G93A^ ALS mice.** (**A**–**C**) SOD1^G93A^ induced activation of genes *C1qa*, which encodes C1q α component, is reduced in SOD/OXR1 mice at Days 90 (P90) and 135 (P135). (**D**) Immunoprecipitation showing induced expression of phosphorylated-STAT3 in the SOD1^G93A^ spinal cord is reduced by overexpression of OXR1 at Day 90. Quantification of p-STAT3, and values are mean ± SEM (*n* = 5 per genotype; *P < 0.05, **P < 0.01, one-way ANOVA with Tukey’s *post hoc* tests). The same blot was reprobed with anti-STAT3. Expression controls for STAT3 and β-actin from total cell lysates are also shown from an independent blot. Complete blot images are shown in Supplementary Fig. 3P–S. (**E** and **G**) SOD1^G93A^ induced activation of *Vim*, a target of STAT3, is significantly reduced in SOD/OXR1 mice at Days 90 and 135. (**A**–**C** and **E**–**G**) Values are mean ± SEM (*n* = 3–5 per genotype; *P < 0.05, **P < 0.01, ***P < 0.001, one-way ANOVA with Tukey’s *post hoc* tests). IP = immunoprecipitation; WB = western blot.

To better understand mechanisms by which OXR1 delays inflammation in the SOD1^G93A^ spinal cord, we further examined activation pathways upstream of the immune response. In particular, the complement pathway was identified by microarray pathway analysis as being significantly activated in SOD/+ but not SOD/OXR1 mice at Day 90 (Supplementary Table 4); due, in part, to genes such as those encoding complement-activating C1q proteins (Lobsiger *et al.*, 2013) being identified as ‘rescued’ by neuronal OXR1 overexpression (Supplementary Table 3). We confirmed upregulation of *C1qa*, *C1qb* and *C1qc* in SOD/+ mice is significantly less pronounced in SOD/OXR1 mice at Days 90 and 135 by quantitative RT-PCR (Fig. 7A–C and Supplementary Fig. 3G–L), suggesting that OXR1 functions upstream of classic complement pathway activation. Furthermore, STAT3, a key regulator of inflammatory response, was identified from pathway analysis as functioning upstream of the ‘rescued’ genes observed at Day 90 (Supplementary Table 5). Interestingly, STAT3 is activated in ALS patients and SOD1^G93A^ mice (Shibata *et al.*, 2009, 2010), thus we determined whether OXR1 overexpression alters levels of phosphorylated STAT3 (p-STAT3) at Day 90 by immunoblotting. Upregulated p-STAT3 in the spinal cord of SOD/+ mice is markedly reduced to wild-type levels under neuronal overexpression of OXR1 (Fig. 7D). Therefore, we next examined expression of two genes, *Vim* and *Serpina3n*, which were identified as ‘rescued’ in the Day 90 microarray, and are downstream targets of the activated STAT3 pathway (Supplementary Tables 3 and 5). At Days 90 and 135, *Vim* and *Serpina3n* are significantly increased in SOD/+ mice, but this upregulation is significantly reduced in SOD/OXR1 mice (Fig. 7E–G and Supplementary Fig. 3M–O), further supporting OXR1 function upstream of the STAT3-activated immune response pathway. Together, these data demonstrate that neuronal OXR1 overexpression is sufficient to decrease pathological neuroinflammation through regulating activation of multiple pathways in the spinal cord of SOD1^G93A^ mice.

## Discussion

Here, we established OXR1 as a novel genetic modifier of ALS, and found that neuron-specific overexpression of OXR1 extends lifespan and delays ALS disease pathogenesis. OXR1 improves motor function, increases motor neuron survival, and protects against oxidative stress in the SOD1^G93A^ spinal cord (Fig. 3). We also demonstrated that OXR1 increases muscle strength, and delays gastrocnemius muscle atrophy and fatigue-resistant EDL muscle fibre changes (Fig. 4). OXR1 is one of only three known proteins that, when overexpressed in neurons, delays motor dysfunction and improves muscle pathology in mutant SOD1^G93A^ mice (Figs 2 and 3) (Storkebaum *et al.*, 2005; Seijffers *et al.*, 2014). Importantly, OXR1 is the first antioxidant that has been shown to increase survival of SOD1^G93A^ mice through specific overexpression in neurons (Turner and Talbot, 2008; Riboldi *et al.*, 2011).

In this study, we conducted microarray analysis to uncover pathways by which OXR1 confers its neuroprotective benefits at Day 90, a time point that we observed is prior to significant motor neuron loss in SOD/+ mice (Figs 3 and 6). We found that overexpression of OXR1 in neurons significantly delays dysregulation of genes that are involved in diverse molecular functions altered in ALS (Olsen *et al.*, 2001; Yoshihara *et al.*, 2002; Chen *et al.*, 2010), including a striking delay in the inflammatory response (Figs 5–7). Neuroinflammation has been inextricably linked to ALS disease progression, and in particular, activated microglia and astrocytes substantially contribute to motor neuron death (Ilieva *et al.*, 2009; Ferraiuolo *et al.*, 2011; Bowerman *et al.*, 2013). Specific deletion of mutant SOD1 from either astrocytes or microglia significantly slows disease progression in SOD1^G93A^ mice, while monoclonal antibody treatment against CD-40L, a T cell surface ligand that activates the immune response, or inhibiting NF-κB activation in microglia significantly extends survival in SOD1^G93A^ mice (Boillee *et al.*, 2006; Yamanaka *et al.*, 2008; Lincecum *et al.*, 2010; Frakes *et al.*, 2014). Moreover, transplantation of SOD1^G93A^ glial-restricted progenitors into spinal cord of wild-type rats induces astrogliosis and microgliosis, motor dysfunction, and motor neuron death, demonstrating non-cell autonomous toxicity in ALS (Papadeas *et al.*, 2011).

Here, we showed that OXR1 influences genes involved in immune activation, including *Ctss* and *Lyz*2, as early as Day 60, a time point prior to disease onset (Fig. 6 and Supplementary Fig. 3). This supports the hypothesis that the delay in neuroinflammation observed in SOD/OXR1 compared to SOD/+ animals is not simply due to a delay in spinal motor neuron death. Indeed, increased motor neuron survival does not always correlate with delayed neuroinflammation. For example, either a 50% decrease in expression of the ephrin receptor *EphA4*, or neuron-specific deletion of the transcription factor *Xbp1*, decreases motor neuron apoptosis in SOD1 mutant mice without altering neuroinflammation (Hetz *et al.*, 2009; Van Hoecke *et al.*, 2012). Therefore our finding that overexpression of OXR1 modulates the immune response prior to significant motor neuron loss during ALS pathogenesis is particularly important.

Furthermore, we demonstrated that OXR1 functions upstream of multiple pathways that activate the immune system, including the complement system and STAT3 pathway. In particular, overexpression of OXR1 delays induction of *C1qa/b/c*, encoding for C1qα-/β-/γ-chains, which initiates the classic complement pathway responsible for mediating the immune response (Fig. 7). Induction of C1q in motor neurons and the global complement pathway in the spinal cord of mutant SOD1 mice has been reported in many studies (Olsen *et al.*, 2001; Perrin *et al.*, 2005; Bonifati and Kishore, 2007; Ferraiuolo *et al.*, 2007; Lobsiger *et al.*, 2007; Lee *et al.*, 2013). While deletion of C1q or C3, a protein central to both the classic and alternative complement pathway, does not affect onset of disease or survival, treatment with a selective C5aR antagonist or loss of C5a receptor CD88 extends survival of mutant SOD1 mice by ∼6% (Woodruff *et al.*, 2008, 2014; Lobsiger *et al.*, 2013). In addition, we found that neuronal OXR1 overexpression delays activation of STAT3 (Fig. 7). In response to proinflammatory cytokines, STAT3, a transcription factor, becomes activated, translocates to the nucleus, and induces expression of additional immune response genes, and activation of STAT3 has been observed in motor neurons and glia cells in the spinal cord of ALS patients and SOD1^G93A^ mice (Shibata *et al.*, 2009, 2010). Moreover, we showed that induction of downstream targets of STAT3, such as *Vim* and *Serpina3n*, is similarly reduced by neuronal OXR1 overexpression (Fig. 7). Vimentin is an intermediate filament that forms inclusions in mutant SOD1 motor neurons, an early pathological hallmark of ALS (Olsen *et al.*, 2001; Yoshihara *et al.*, 2002; Perrin *et al.*, 2005; Ferraiuolo *et al.*, 2007). Similarly, ERPINA3N accumulates along these neurofilamentous conglomerates, and the imbalance of serine proteases and their respective inhibitors during oxidative injury may contribute to the formation of ALS motor neuron inclusions (Chou *et al.*, 1998). Together, this evidence suggest that OXR1 functions upstream of multiple neuroinflammatory pathways during ALS pathogenesis, and that addressing multiple pathogenic mechanisms may be required to improve survival due to compensatory pathways.

Cross-talk between neuroinflammation and oxidative stress during ALS pathogenesis has been previously hypothesized (Barber and Shaw, 2010). Damaged motor neurons release reactive oxygen species, inducing oxidation and reducing glutamate uptake in neighbouring astrocytes, which subsequently leads to a cyclical process by which increased extracellular glutamate levels and excitotoxicity further damage motor neurons (Barber and Shaw, 2010). Reactive oxygen species activate glial cells, which can subsequently generate more reactive oxygen species, reactive nitrogen species, and proinflammatory mediators, and thereby activating more glial cells, all of which lead to further motor neuron injury (Barber and Shaw, 2010). Furthermore, mutant SOD1 in microglia disrupts regulation of NADPH oxidase activity through persistent activation of RAC1, resulting in prolonged production of reactive oxygen species, which promote motor neuron apoptosis (Harraz *et al.*, 2008; Marchetto *et al.*, 2008). While exact mechanisms by which OXR1 regulates the immune response remain elusive, OXR1 protects against oxidative stress-induced apoptosis (Oliver *et al.*, 2011), and loss of OXR1 results in astrogliosis and microgliosis in the cerebellum and spinal cord (Supplementary Fig. 5). Therefore, we hypothesize that OXR1 mediates cross-talk between injured neurons and toxic mutant SOD1 microglia and astrocytes by (i) conferring protection against increasing intracellular levels of reactive oxygen species, reducing the release of reactive oxygen species from damaged neurons, and thereby decreasing glial cell activation; and (ii) conferring protection against extracellular levels of reactive oxygen species released by activated glia, which subsequently further extends survival of motor neurons, and delays activation of additional glia.

Our previous data demonstrating OXR1 overexpression in end-stage ALS spinal cord biopsies (Oliver *et al.*, 2011) suggest that the CNS is able to induce expression of the protein as part of a stress response. This conclusion is in-line with the initial studies of OXR1 that demonstrated induction of the protein under heat or oxidative stress (Volkert *et al.*, 2000; Elliott and Volkert, 2004; Oliver *et al.*, 2011), in addition to *in vivo* studies in which *Oxr1* expression is induced under hypoxia in the mouse retina (Natoli *et al.*, 2008). Conversely, there are several lines of evidence that the loss or reduction of OXR1 is detrimental to the CNS *in vivo*; not only in the mouse (Oliver *et al.*, 2011), but also in the fly (Fischer *et al.*, 2001) and nematode worm (Sanada *et al.*, 2014). Most recently, OXR1 knockdown studies have suggested that OXR1 modulates antioxidant pathways via the cyclin-dependent kinase inhibitor 1A (p21) protein and nuclear factor (erthyroid-derived 2)-like 2 (Nrf2, now known as NFE2L2) (Yang *et al.*, 2014), although the molecular mechanisms are still unknown. Indeed, the *OXR1* gene does not seem to contain a positionally conserved antioxidant response element (ARE), suggesting that OXR1 acts upstream of Nrf2-related pathways (Wang *et al.*, 2007). In summary, it is clear that the levels of OXR1 are critical for neuronal survival, and it will be important in the future to examine the role of this protein within the antioxidant defence and immune response pathways.

Finally, a meta-analysis of therapeutic strategies found that drugs targeting inflammation prior to onset of symptoms and antioxidant pathways at symptom onset are most effective in prolonging survival in mutant SOD1 mice (Benatar, 2007). Here, we found that overexpression of antioxidant OXR1 in neurons before onset of symptoms modifies SOD1-mediated ALS and delays inflammatory response, and presented evidence that a novel neuronal antioxidant modulates the cross-talk between neuroinflammation and oxidative stress during neurodegenerative pathogenesis. Moreover, neuronal OXR1 overexpression *in vivo* has no overt adverse effects (Fig. 1 and Supplementary Fig. 2), and does not detrimentally alter the spinal cord transcriptome (Supplementary Table 2). Taken together, we demonstrated the potential for OXR1 to serve as a therapeutic target for ALS patients.

## Abbreviation

ALS: amyotrophic lateral sclerosis
